# Development and clinical validation of an artificial intelligence based model for thyroid nodule malignancy risk assessment using C-TIRADS guidelines

**DOI:** 10.3389/fmed.2026.1877809

**Published:** 2026-07-15

**Authors:** Rongzhou Ye, Yao Liu, Xiuming Wu, Kangjian Wang

**Affiliations:** 1 Department of General Practice, Quanzhou First Hospital Affiliated to Fujian Medical University, Quanzhou, China; 2 College of Engineering, Huaqiao University, Quanzhou, China; 3 Department of Ultrasound Medicine, Quanzhou First Hospital Affiliated to Fujian Medical University, Quanzhou, China; 4 Department of Ultrasound Medicine, Zhangzhou Affiliated Hospital of Fujian Medical University, Zhangzhou, China

**Keywords:** artificial intelligence, C-TIRADS guidelines, deep learning, risk scoring model, thyroid nodule

## Abstract

**Background:**

Thyroid nodules refer to discrete lesions within the thyroid gland, caused by abnormal proliferation of thyroid cells and local growth. Thyroid ultrasound imaging is a non-invasive, widely used technique primarily employed to assess the benign or malignant nature of nodules, the extent of invasion into adjacent tissues, and lymph node metastasis. Early ultrasound diagnosis can help reduce the incidence of thyroid cancer. However, the diagnostic results from ultrasound examinations are often subjective, labor-intensive, and highly dependent on the clinical experience of the ultrasound physician.

**Methods:**

We developed a C-TIRADS-guided computer-aided diagnostic framework consisting of a nodule detection module, a C-TIRADS feature classification module, and a rule-based risk scoring module. Thyroid nodules were first localized using the detection network and then classified according to key C-TIRADS ultrasound features, including composition, echogenicity, margin, shape, and echogenic foci. The predicted feature scores were aggregated to determine the benign or malignant risk category. The model was trained using task-specific datasets and clinically validated on an independent cohort of 303 thyroid nodules/images from 284 patients.

**Results:**

In the independent clinical validation set, the AI model achieved an overall accuracy of 0.862 [95% CI, 0.822–0.901]. Physician accuracy was 0.705 [95% CI, 0.657–0.756] without AI assistance and increased to 0.845 [95% CI, 0.802–0.884] with AI assistance. These findings suggest the potential value of the proposed system as a C-TIRADS-guided decision-support tool.

**Conclusion:**

The proposed C-TIRADS-guided framework can localize thyroid nodules, classify guideline-defined ultrasound features, and provide standardized malignancy risk stratification. The model may assist physicians in thyroid ultrasound interpretation; however, larger blinded multicenter studies are required before routine clinical application.

## 1 Introduction

A thyroid nodule refers to a discrete lesion within the thyroid gland, caused by abnormal and focal growth of thyroid cells. Thyroid nodules are common, and generally asymptomatic. Due to the widespread use of diagnostic imaging technologies, thyroid nodules are frequently detected. The prevalence of thyroid nodules in the general population is as high as 68%, with approximately 7 to 15% being malignant (1).

Ultrasound is the optimal imaging modality for evaluating nodules (2). It is particularly effective for the initial assessment of two types of thyroid nodules: palpable nodules and those detected through other imaging studies. Ultrasound has high sensitivity in identifying the number and characteristics of thyroid nodules, as well as high-risk features for malignancy, such as microcalcifications or irregular nodule shapes or margins. The presence of high-risk features, especially when two or more such features are found together, significantly increases the risk of thyroid cancer. Ultrasound can identify up to 50% of nodules, with 90% of these diagnosed as benign (3, 4). In terms of diagnosis and treatment, benign thyroid nodules are mostly managed with follow-up observation, while malignant thyroid tumors are typically treated with surgery. However, in practice, thyroid nodules exhibit significant heterogeneity, with heterogeneous internal components, and the ultrasound images of benign nodules and malignant tumors often overlap. Additionally, ultrasound images themselves often contain artifacts and noise, which affects the accuracy and consistency of physicians’ assessments of thyroid ultrasound images (5). Currently, many researchers are focused on developing non-subjective methods, such as artificial intelligence models, to extract a large amount of untapped digital information from images, aiming to address issues such as varying professional levels, high subjectivity, and poor reproducibility in ultrasound examinations.

With the development of artificial intelligence (AI) technology, computer- aided detection (CAD) has become a feasible and cost-effective solution. It utilizes computer technology to analyze ultrasound medical images, automatically identify standard cross-sectional images during the ultrasound examination process, and assess the disease risk of the corresponding organs (6, 7). For in- stance, Yang et al. proposed an improved YOLOv5 neural network to assist physicians in diagnosing thyroid cancer. The network includes a Coordinate Attention (CA) module and a Label Smoothing Regularization (LSR) module, where the CA module enables the network to extract positional information. The improved neural network is capable of accurately identifying lesion area and nodule type within 8.4 ms (8). Manh et al. proposed a novel deep learning (DL) framework called Multi-Attribute Attention Network (MAA-Net), designed to simulate the clinical diagnostic process. The model learns to predict nodule attributes and infers its malignancy based on these clinically relevant features. By employing a multi-attention mechanism, it generates customized focus to enhance the performance of each task and improve the diagnostic accuracy for malignant tumors (9). Despite the progress made by these methods in assisting thyroid nodule diagnosis, existing computer-aided thyroid ultrasound diagnostic technologies still face numerous challenges. Firstly, constructing a large dataset with complete pathological results requires substantial effort. In clinical practice, physicians typically do not recommend fine-needle aspiration biopsy (FNAB) for benign nodules, making it a lengthy and challenging task to obtain pathological results for images from all patients. Secondly, many studies focus on the characteristics of benign and malignant nodules when collecting nodule images. However, due to the similarity of features within nodules detected in a single examination, it is difficult to ensure that the collected data comprehensively covers all benign and malignant characteristics. Finally, ultra- sound examinations are real-time and fast processes, necessitating the use of a lightweight and robust network to assist physicians in diagnosis. Furthermore, integrating clinical prior knowledge and providing detailed evaluation information can enhance the network’s interpretability, aiding physicians in making more precise decisions. For malignant nodules, avoiding misdiagnosis is critical to prevent missing early treatment opportunities.

This study provides practical and feasible recommendations for addressing several issues. First, regarding data collection, this study is based on the” Chinese Thyroid Imaging Reporting and Data System (C-TIRADS)” guidelines used by ultrasound physicians in clinical practice. The study focuses on five categories with scoring: morphology, margin, structure, echogenicity, and focal hyperechoic areas, therefore, this study constructed task-specific datasets based on the C-TIRADS guideline for nodule detection, ultrasound feature classification, and independent clinical validation. Each image contains specific features according to the C-TIRADS standards. Second, the model construction. This study proposes a deep learning model based on the attention mechanism, which is divided into two parts. The first part aims to enhance the model’s ability to identify thyroid nodules by introducing a lightweight deep learning model. This model consists of three networks: feature extraction, feature aggregation, and nodule recognition. The feature extraction network extracts nodules from ultra- sound images, and the feature fusion network improves the accuracy of nodule feature recognition. The final recognition network uses nodule features to locate the nodules. The second part aims to improve the model’s interpretability for physicians. This study integrates the 2020 Chinese Thyroid Nodule Ultrasound Malignancy Risk Stratification Guidelines: C-TIRADS (10) published by the Chinese Medical Association’s Ultrasound Medicine Branch and the Chinese Thyroid and Breast Ultrasound AI Alliance in the Chinese Journal of Ultra- sound. It uses a classification model to analyze multiple features of nodules, including structure, margin, shape, echogenicity, and focal hyperechoic areas. By aggregating the scores of these feature categories, a more precise judgment of nodule malignancy risk can be made. In the clinical validation set, the AI model achieved a precision, recall, and F1 score of 0.710, 0.936, and 0.807 for benign nodules, respectively, and 0.967, 0.827, and 0.891 for malignant nodules. Physician accuracy increased from 0.705 without AI assistance to 0.845 with AI assistance. The main contributions of this study are summarized as follows:

- A C-TIRADS-guided decision-support framework was developed for thyroid nodule localization, ultrasound feature classification, and guideline-based malignancy risk stratification.
- A lightweight thyroid nodule detection module was constructed to localize thyroid nodules and provide cropped regions of interest for subsequent feature classification.
- Task-specific datasets were constructed for nodule detection, C-TIRADS feature classification, and independent clinical validation, supporting a structured evaluation of the proposed framework.

The structure of this paper is as follows: Section 2 reviews relevant studies on deep learning and machine learning methods in the medical field; Section 3 describes the proposed framework for thyroid nodule detection and classification; Section 4 presents the details of the thyroid ultrasound image dataset, experimental setup, and final experimental results; finally, Section 5 provides the discussion and conclusion.

## 2 Related work

In recent years, artificial intelligence (AI) and deep learning have emerged as transformative tools in the medical field, driving significant advancements in automated disease prediction and clinical risk assessment. For instance, advanced deep learning frameworks have been extensively leveraged to analyze complex clinical data, substantially improving the accuracy of disease prognosis and diagnostic workflows (11). Concurrently, the integration of vision transformers and optimized computing architectures has revolutionized medical image analysis, allowing models to capture fine-grained patterns and long-range dependencies that traditional convolutional neural networks might miss (12). These general-purpose medical AI methodologies have established a robust foundation for specialized tasks, validating the feasibility of utilizing deep models to mitigate clinician subjectivity and enhance diagnostic consistency across various clinical settings (13). Building upon these foundational breakthroughs, the application of machine learning (ML) and deep learning (DL) has witnessed profound success within the specific realm of medical imaging. Driven by the rapid expansion of computational power and big data, the accuracy and efficiency of medical image analysis have been notably improved. Among various imaging modalities, the automated diagnostic evaluation of thyroid ultrasound scans has attracted substantial research interest. To enhance the recognition and detection of thyroid nodules in ultrasound images, Song et al. proposed a machine learning approach to perform this task and developed a multi-task cascading convolutional neural network framework (MC-CNN) to leverage the contextual information of thyroid nodules (14). To overcome the inherent limitations of small medical image datasets and the time-consuming process of obtaining lesion annotations, Yang et al. proposed a multi-task cascaded deep learning model (MCDLM). This model integrates various domain knowledge (DK) from radiologists and utilizes multimodal ultrasound images for the automated diagnosis of thyroid nodules (15).

The medical images contain a wealth of clinical prior knowledge, and considering this knowledge during medical image research can help improve the performance of models. The risk stratification guidelines for thyroid nodules are based on certain well-known ultrasound features of the nodules, but their application remains subjective, as interpretation heavily relies on the physician’s experience. These guidelines classify nodules based on a limited set of subfeatures of ultrasound signs. Ozturk et al. proposed an artificial intelligence- based method to examine the relationships of various ultrasound (US) features in the differential diagnosis of nodules to overcome these limitations. They innovatively used a genetic algorithm (GA) to train an adaptive network-based fuzzy inference system (ANFIS) to differentiate malignant from benign thyroid nodules (16). Due to the significant uncertainty in the malignancy risk of TI- RADS class 4 thyroid nodules, the risk ranges from 5 to 80%. Class 4 thyroid nodules are further subdivided into three subtypes: 4a, 4b, and 4c. Accurate classification of these subtypes is of great clinical significance. Lu et al. pro- posed a method based on ultrasonographic features and a multi-kernel learning algorithm for classifying TI-RADS class 4 thyroid nodules (17). The diagnosis of thyroid cancer and the differentiation between benign and malignant thyroid nodules by primary radiologists pose significant challenges. Jin et al. proposed a computer-aided diagnosis (CAD) system based on the Thyroid Imaging Re- porting and Data System (TI-RADS). This system analyzes ultrasound images to distinguish between benign and malignant thyroid nodules, aiming to enhance the diagnostic capabilities of primary radiologists. The system employs an improved TI-RADS, developed using a convolutional neural network (CNN), for the CAD system (18). To provide reasoning for the classification of thyroid nodules malignancy in computer-aided diagnosis (CAD) systems, Manh et al. proposed a novel deep learning (DL) framework called Multi-Attribute Attention Network (MAA-Net), designed to simulate the clinical diagnostic process. The model learns to predict nodule attributes and infers malignancy based on these clinically relevant features. A multi-attention mechanism is employed to generate customized attention, enhancing the performance of each task and optimizing the accuracy of malignancy diagnosis (9).

These methods have successfully assisted physicians in diagnosing thyroid nodules. However, there are still areas that require improvement. Specifically, some traditional computer-aided diagnostic (CAD) methods rely on manually designed features, which may fail to comprehensively capture the complexity and details of the nodules. In addition, some traditional CAD methods may rely solely on imaging features without adequately incorporating clinical diagnostic standards, thus limiting their clinical applicability. Therefore, this study aimed to design a C-TIRADS-guided computer-aided method that can rapidly localize thyroid nodules and provide standardized ultrasound feature-based malignancy risk stratification, thereby assisting ultrasound physicians in thyroid nodule assessment.

## 3 Study design

The dataset was constructed from thyroid ultrasound images collected from participating hospitals. To support different model-development tasks, the data were organized into three task-specific subsets: Training Set A for thyroid nodule detection, Training Set B for C-TIRADS feature classification, and Test Set C for clinical validation.

Training Set A was used for thyroid nodule detection and contained 1,921 thyroid ultrasound images after quality control. Training Set B was used for C-TIRADS feature classification and contained 6,469 labeled samples, including 2,783 samples for composition, 1,163 for echogenicity, 1,742 for margin, and 781 for echogenic foci. Test Set C was used as the independent clinical validation set and contained 303 thyroid nodules/images from 284 patients, including 94 benign and 209 malignant nodules. Patients in the clinical validation set did not overlap with those in the training datasets. All data were de-identified before model development and evaluation.

All collected images were converted from DICOM format to JPEG format. These images were acquired using ultrasound equipment from various manufacturers, including GE Healthcare, Philips, Siemens, Canon, Samsung, Esaote, Mindray, SonoScape, Aloka, BK Medical, Supersonic, Vinno, and Hitachi. To ensure image quality, all thyroid images were screened by professional radiologists with at least 3 years of experience in ultrasound diagnostics. Images were excluded if there were discrepancies between the image and the pathological report regarding the nodule location. Before quality control, Training Set A included 1,997 images from 1,357 patients for thyroid nodule detection. After excluding 76 images because of poor image quality, 1,921 images remained. Training Set B included 6,469 labeled samples from 5,429 patients for C-TIRADS feature classification after quality control. Before quality control, Test Set C included 317 representative ultrasound images from 284 adult patients. After excluding 14 images that did not meet the quality-control criteria, 303 thyroid nodules/images remained for clinical validation, including 94 benign and 209 malignant nodules. All data were de-identified before model development and evaluation. Detailed data specifications are provided in Table 1. Table 1 summarizes the final included samples in each task-specific dataset. The corresponding patient numbers and quality-control process are described in the preceding paragraph.

**Table 1:** Distribution of the datasets.

Datasets	Categories	Quantity
Training Set A	Thyroid nodules	1921
Training Set B	Composition	2,783
	Echogenicity	1,163
	Margin	1742
	Echogenic foci	781
Test set C	Benign	94
	Malignant	209

**Figure 1:**
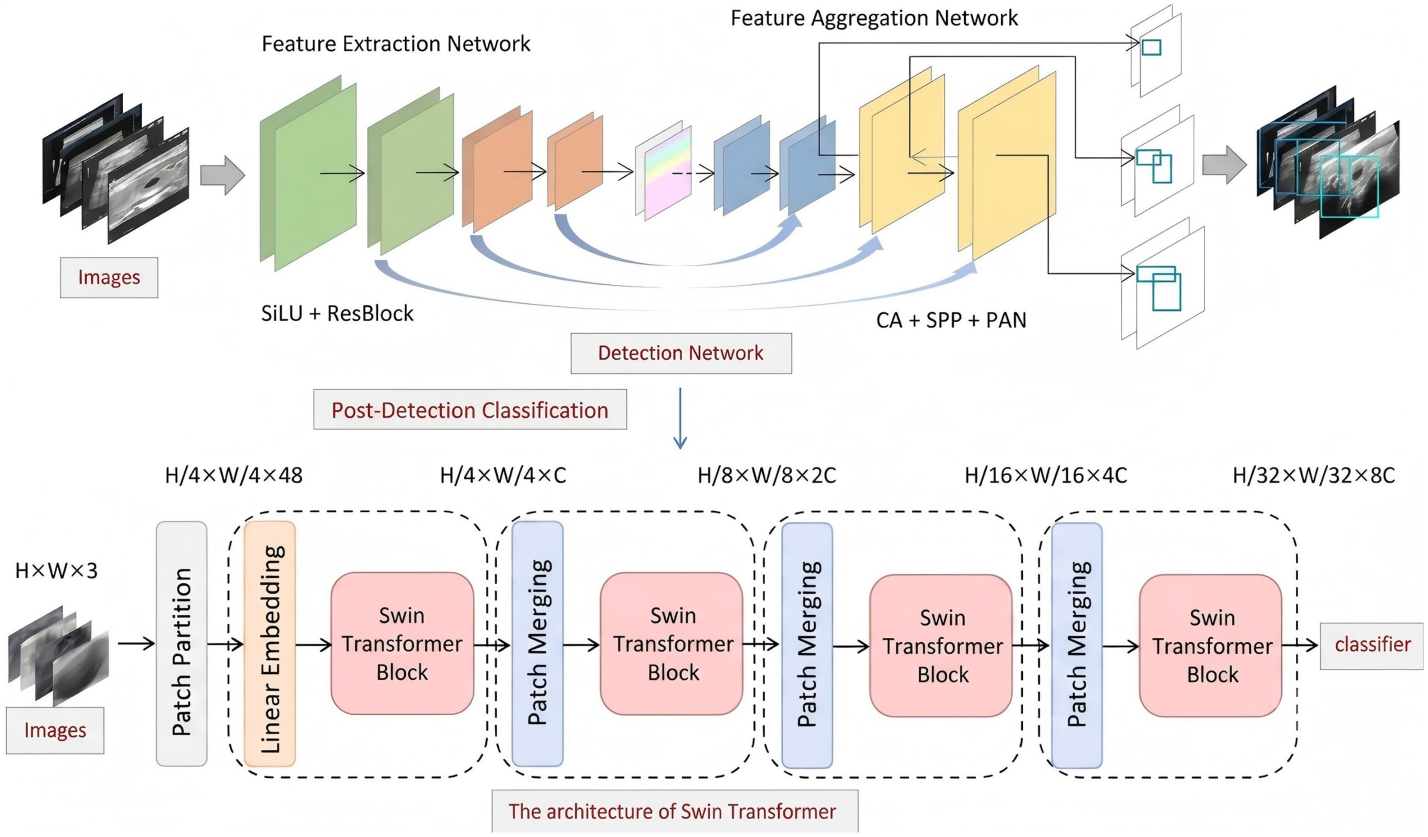
Experimental overview: data from three centers were used. A scoring model was trained to differentiate thyroid nodules. First, a detection model was trained to identify thyroid nodules, followed by a classification model for scoring.

The thyroid nodule risk scoring model was designed to support C-TIRADS-guided malignancy risk stratification from thyroid ultrasound images. The architecture of the model is primarily divided into two core networks: the thyroid nodule detection network and the thyroid nodule classification network (Figure 1). Initially, thyroid nodule ultrasound images are subjected to preliminary identification by the detection network. The identified nodules are then cropped and input into the classification network. This network classifies the nodules based on their features, including structure, echogenicity, shape, margin, and echogenic foci. The shape feature of the nodules is directly calculated using the aspect ratio of the bounding box generated by the detection network. Finally, a scoring discriminator aggregates the scores of the various feature categories to generate a diagnostic report, which is annotated on the original image. All methods were performed in accordance with the relevant guidelines and regulations.

In computer vision, the feature extraction network, feature aggregation net- work, and Nodule Recognition Network are critical components of a thyroid nodule detection networks. The feature extraction network employs Convolutional Neural Networks (CNNs) to extract key features from input images, such as edges, textures, and shapes. The feature aggregation network integrates these features at different scales and levels, thereby enhancing the model’s robustness and discriminative power. Finally, the classification detection network utilizes the integrated features for image classification and object detection, precisely localizing thyroid nodules.

The feature extraction network in this study adopts an improved version of CSPDarkNet (19) as its foundational architecture, which is a lightweight convolutional neural network. It utilizes CBS modules and bottleneck-structured residual blocks for feature compression and downsampling, progressively reducing input images from 640 × 640 pixels to 20 × 20 pixels while expanding channel numbers to effectively extract multi-scale features. The network employs SiLU activation functions (20) and simplified ResBlock stacking, optimizing the model’s lightweight design to extract crucial features from ultrasound images, thereby providing high-quality inputs for subsequent detection and enhancing the precision and richness of nodule recognition.

The feature fusion network includes Coordinate Attention (CA) modules (21), Spatial Pyramid Pooling (SPP) modules (22), and Path Aggregation Net- work (PAN) modules (23). The CA module generates feature maps with spatial attention weights, the SPP module mitigates the decay of target positional in- formation through multi-scale pooling, and the PAN module enhances semantic information in a top-down manner, combined with an FPN structure to fuse multi-scale feature maps, thereby improving the accuracy and robustness of thyroid nodule recognition. The network design emphasizes computational efficiency and flexibility, making it suitable for various clinical environments and devices.

The nodule recognition network effectively detects and locates nodules of different sizes and shapes through multi-scale strategies and precise feature ex- traction. Initial anchor boxes are generated using k-means clustering, optimized via genetic algorithms, and the CIoU loss function is employed to enhance localization accuracy (24). The model performance is evaluated using indicators such as specificity and sensitivity (recall rate), providing important support for the automation and clinical application of thyroid nodule detection.

**Figure 2:**
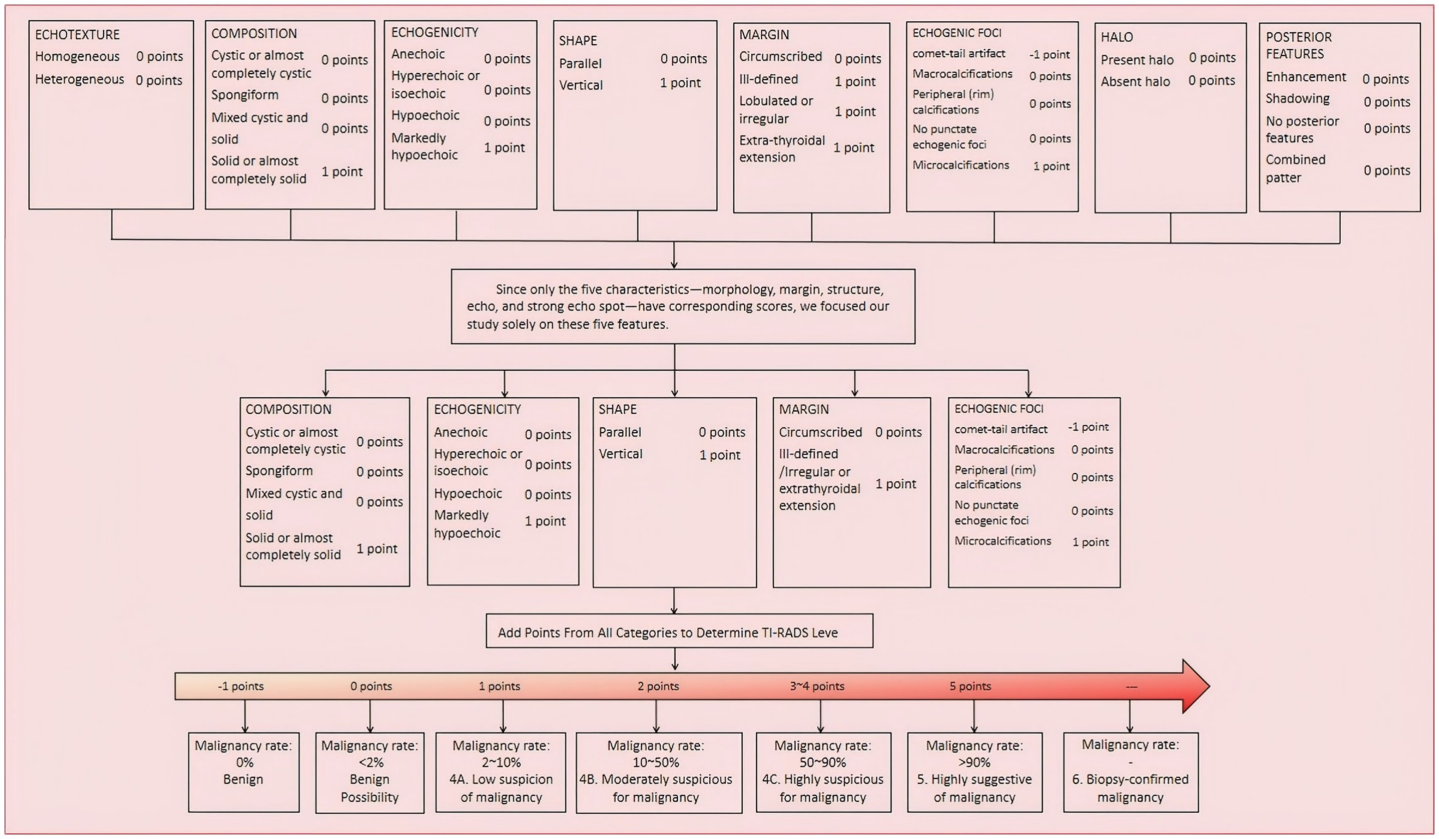
2020 Chinese guidelines for ultrasound risk stratification of thyroid nodules: C-TIRADS.

The goal of constructing a thyroid nodule classification network is to pro- vide clinicians with a more precise risk assessment of the benign or malignant nature of thyroid nodules. We employed the C-TIRADS guidelines, which thoroughly categorize nodules into nine characteristics: location, shape, margin, halo, structure, echogenicity, echogenic texture, echogenic foci, and posterior acoustic features (25). In our final risk assessment, we primarily focus on five categories: shape, margin, structure, echogenicity, and echogenic foci (Figure 2). According to the C-TIRADS guidelines, shape is categorized into vertical and horizontal types, which can be determined through the detection frame from the previous stage; thus, no additional data collection is required at this stage. The margin characteristic is divided into smooth, irregular, blurred, and extrathyroidal extension categories, with the latter three categories each scoring 1 point. After consulting with clinical experts, we simplified the margin categories into smooth and non-smooth. The structure characteristic is divided into solid, predominantly solid, predominantly cystic, cystic, and spongiform according to C-TIRADS, with solid nodules scoring 1 point. For data collection, we grouped solid and predominantly solid nodules into one category, predominantly cystic and cystic nodules into another, and added a mixed cystic-solid category. Echogenicity is classified into hyperechoic, isoechoic, hypoechoic, markedly hypoechoic, and anechoic categories, with markedly hypoechoic nodules scoring 1 point. Under clinical guidance, we merged hyperechoic and isoechoic nodules into a single category. As for echogenic foci, the C-TIRADS guidelines clas- sify them into punctate echogenic foci (microcalcifications), comet tail artifacts, macrocalcifications, peripheral calcifications, and no echogenic foci, with comettail artifacts scoring −1 point as a benign indicator and punctate echogenic foci scoring 1 point as a malignant indicator.

Model calculates the total risk score of a nodule by summing the scores of the five aforementioned features. According to Figure 2, each score corresponds to a specific malignancy rate and C-TIRADS classification (23): A comet tail, which is a benign indicator, can reduce the score to −1, resulting in a 0% malignancy rate. When the score is 0, the malignancy rate is less than 2%, and the C-TIRADS classification is 3, indicating a benign likelihood. A score of 1 cor- responds to a malignancy rate of 2 to 10% and is classified as 4A, indicating low suspicion of malignancy. A score of 2 corresponds to a malignancy rate of 10 to 50% and is classified as 4B, indicating moderate suspicion of malignancy. Scores of 3 to 4 correspond to a malignancy rate of 50 to 90% and are classified as 4C, indicating high suspicion of malignancy. A score of 5 corresponds to a malignancy rate greater than 90% and is classified as 5, indicating high likelihood of malignancy. After discussion with clinical physicians, a model-generated C-TIRADS score of 3 or higher was categorized as high-risk/suspicious for malignancy, whereas a score below 3 was categorized as low-risk/likely benign. This threshold was used to convert the guideline-based risk score into a binary risk category for clinical validation. It should be noted that the classification module was trained to recognize C-TIRADS-defined ultrasound features, and the final score was generated according to the guideline-based scoring rules. Therefore, the proposed system should be interpreted as a C-TIRADS-guided risk stratification tool rather than a direct substitute for histopathological diagnosis. This decision criterion aims to provide clinicians with a clearer basis for risk assessment.

To delve deeper into the characteristics of nodules, we have constructed a nodule classification network that incorporates clinical prior knowledge. Currently, mainstream classification networks include convolutional neural networks (CNN) such as ResNet and ShuffleNet, as well as attention mechanism-based networks like Vision Transformer (ViT) and Swin Transformer (26). Swin Trans- former, designed by Microsoft Research Asia, is an efficient image classification model that optimizes the Vision Transformer. This model partitions the input feature map through Patch Partition and Linear Embedding operations, employing a local attention mechanism to significantly reduce computational load. Additionally, the Shifted Window method enables the model to better capture multi-scale features, thereby enhancing classification performance.

## 4 Experiment analysis

### 4.1 Dataset settings

This study was conducted in collaboration with the Second Affiliated Hospital of Fujian Medical University, Zhangzhou Municipal Hospital, and Quanzhou First Hospital. The inclusion criteria were as follows: (1) patients were at least 18 years old; (2) thyroid nodules were confirmed by ultrasound examination; and (3) for the clinical validation set, each nodule had a definitive benign or malignant reference diagnosis based on surgical pathology or fine-needle aspiration cytology classified as Bethesda II or VI. Images with severe artifacts, low resolution, inconsistent lesion-location information, or incomplete annotations were excluded.

After quality control, three task-specific datasets were included. Training Set A, used for thyroid nodule detection, contained 1,921 ultrasound images. Training Set B, used for C-TIRADS feature classification, contained 6,469 labeled samples, including 2,783 for composition, 1,163 for echogenicity, 1,742 for margin, and 781 for echogenic foci. Test Set C, used for clinical validation, contained 303 thyroid nodules/images from 284 patients after excluding 14 images that did not meet the quality-control criteria. Among these 303 nodules/images, 94 were benign and 209 were malignant. The training datasets and clinical validation dataset were separated at the patient level to avoid data leakage.

### 4.2 Reference standard and image annotation

For the nodule detection task, thyroid nodule bounding boxes were manually annotated by trained radiologists and reviewed by senior ultrasound physicians. These bounding-box annotations were used as the reference labels for Training Set A.

For the C-TIRADS feature classification task, labels for composition, echogenicity, margin, echogenic foci, and shape were assigned according to the 2020\u00B0C-TIRADS guideline by experienced ultrasound physicians. Disagreements in feature annotation were resolved by consensus. These feature labels, rather than benign/malignant pathological labels, were used to train the C-TIRADS feature classification module in Training Set B.

For the independent clinical validation set, the benign or malignant reference standard was based on surgical pathology or fine-needle aspiration cytology classified as Bethesda II or VI. This reference diagnosis was independent of both the model-generated C-TIRADS score and the physicians’ C-TIRADS-based assessment. Therefore, the final benign/malignant performance of the proposed system was evaluated against pathology/cytology-based reference diagnoses rather than against physician C-TIRADS scoring alone.

### 4.3 Experimental details

In this study, we employed a series of strategies and techniques to optimize and evaluate the thyroid nodule detection and classification model. First, we adjusted and normalized the input images to ensure a uniform size of 640 × 640 pixels for easier model processing. To mitigate the risk of overfitting, we implemented data augmentation techniques, including random horizontal flipping, translation, and scaling, allowing each image in a training iteration to undergo one of these operations or remain unchanged (27). Additionally, we used the stochastic gradient descent (SGD (28)) algorithm for model training, with an initial learning rate of 0.01, a decay rate of 0.98, and a momentum parameter of0.96 to accelerate convergence. Each batch processed 32 images, and after 300 training iterations, we saved the model parameters that performed best on the validation set.

We used pretrained network weights from the ImageNet dataset as the starting point for model initialization. To ensure consistency during the training process, we applied the same training parameters to each network branch. The optimization algorithm used was stochastic gradient descent (SGD), combined with a cross-entropy loss function to adjust network weights. The initial learning rate was set to 0.01 and was reduced by a factor of 10 every 100 epochs until reaching a minimum learning rate of 0.0001. To suppress overfitting, we incorporated batch normalization into the network and set the weight decay rate to 0.0005. Additionally, we set the batch size to 8. To ensure the reproducibility of the experimental results, all experiments were conducted in a unified experimental environment configured with the Windows 10 operating system. The computer hardware included an Intel(R) Core(TM) i7-11700 CPU, 16 GB of memory, and an NVIDIA GeForce GTX 3060 GPU with 12 GB of video memory. The software environment was Python 3.7, and the deep learning framework used was PyTorch 1.10.1.

During the model evaluation phase, we used various performance metrics to evaluate the classification effect. In object detection, true positives (TP) refer to the number of positive images correctly identified by the model; false negatives (FN) refer to the number of positive images that the model failed to identify; false positives (FP) refer to the number of negative images that the model incorrectly identified as positive images; and true negatives (TN) refer to the number of images correctly identified as background. These metrics allow us to calculate precision, specificity, F1 score, accuracy, and sensitivity (recall) (29). Accuracy directly reflects the ability of the model to correctly identify positive and negative samples.

The evaluation process consisted of three parts: nodule detection model evaluation, C-TIRADS feature classification model evaluation, and exploratory clinical validation. In the nodule detection evaluation, the proposed model was compared with mainstream object detection models. In the C-TIRADS feature classification evaluation, the classification module was compared with mainstream image classification networks under the same available training setting. In the clinical validation phase, the validation images were randomly shuffled and anonymized before physician assessment. Two ultrasound physicians who had completed standardized thyroid ultrasound and C-TIRADS training independently reviewed the original images without access to pathological results, AI predictions, or the other physician’s assessment. Detailed information regarding the physicians’ exact years of experience and professional titles was not available for retrospective reporting. Each lesion was evaluated according to five C-TIRADS categories, including composition, echogenicity, shape, margin, and echogenic foci. Disagreements were resolved by consensus. In the AI-assisted reading phase, the same physicians re-evaluated the images with access to the AI-generated C-TIRADS feature predictions and risk category. Because the physicians had been informed of the model performance before the AI-assisted reading, expectation bias may have been introduced. Therefore, the AI-assisted results were interpreted as an exploratory assessment of the potential value of AI assistance rather than definitive evidence of clinical effectiveness. Future prospective validation should adopt blinded, randomized multi-reader multi-case designs with appropriate washout intervals or parallel reading arms. The available implementation details were reported as completely as possible based on the retained experimental records. Some hyperparameter-level details, including exact anchor-clustering parameters, random seed, CUDA version, and inference-time logs, were not fully preserved and therefore could not be reported in this revision.

### 4.4 Statistical analysis

Statistical analyses were performed based on the available validation results. Diagnostic performance was summarized using accuracy, precision/positive predictive value, recall/sensitivity, and F1 score. The 95% confidence intervals for proportion-based metrics were reported where available. Because only binary classification results and aggregate diagnostic metrics were available in the current study, receiver operating characteristic analysis, calibration analysis, and decision-curve analysis were not performed. Therefore, the clinical validation results should be interpreted as descriptive and exploratory. No formal hypothesis testing was performed because only binary classification results and aggregate diagnostic metrics were available. Therefore, the clinical validation results were interpreted descriptively.

### 4.5 Experimental results analysis

**Figure 3:**
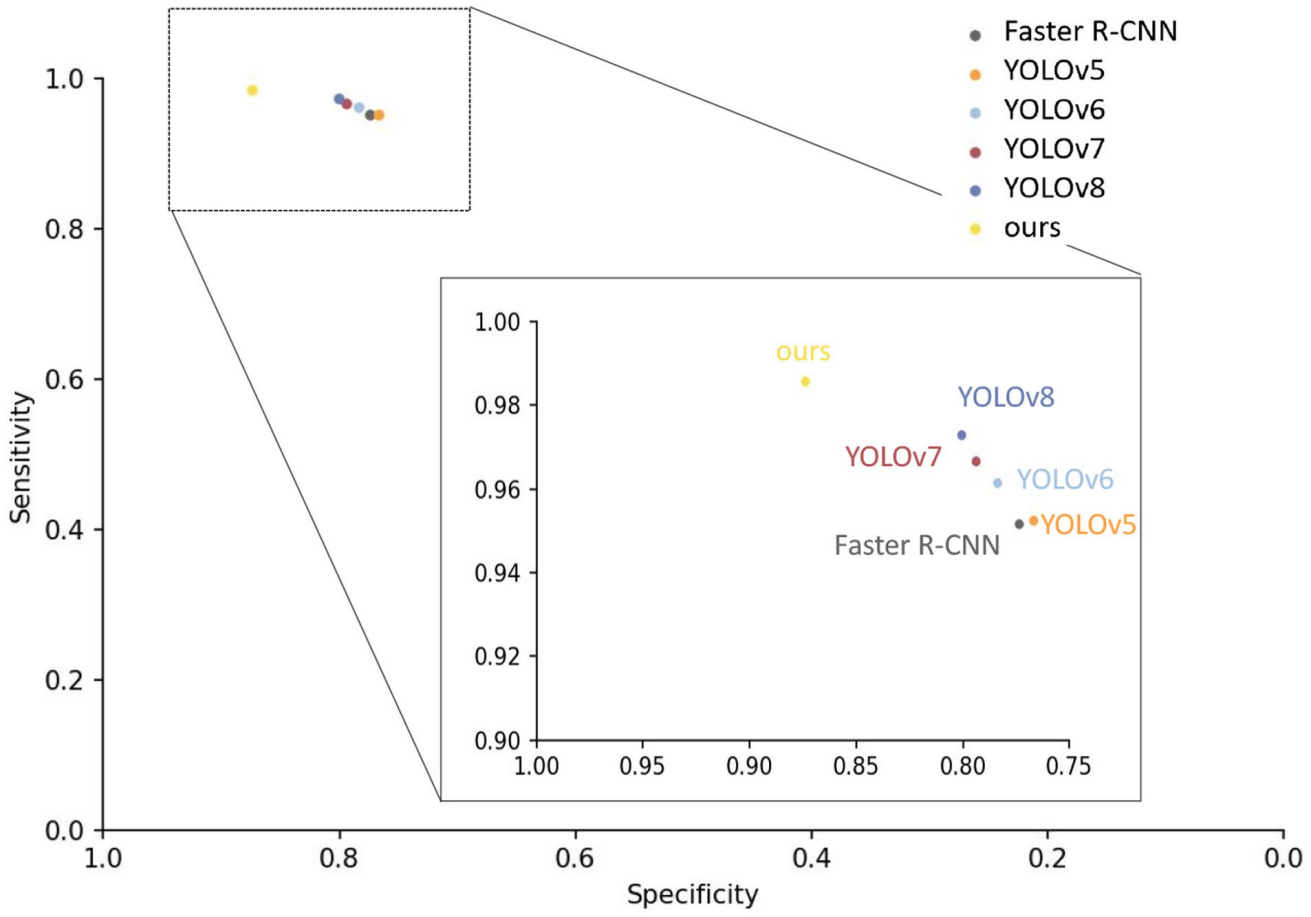
Comparison results of mainstream target detection models. Comparison of the performance of various target detection models in terms of sensitivity and specificity.

Figure 3 shows the performance comparison of various target detection models in terms of sensitivity and specificity. Compared with the YOLO series and Faster R-CNN (30), the proposed model performs best in high sensitivity and high specificity areas, indicating that it has excellent overall performance in correctly identifying positive and negative samples. YOLOv8 (31) performs well in specificity, while YOLOv7 (32) and YOLOv6 (33) has an advantage in sensitivity. Faster R-CNN and YOLOv5 (34) have weaker overall performance, with low sensitivity and specificity. This result shows that the proposed model is suitable for scenarios with high detection accuracy requirements.

**Figure 4:**
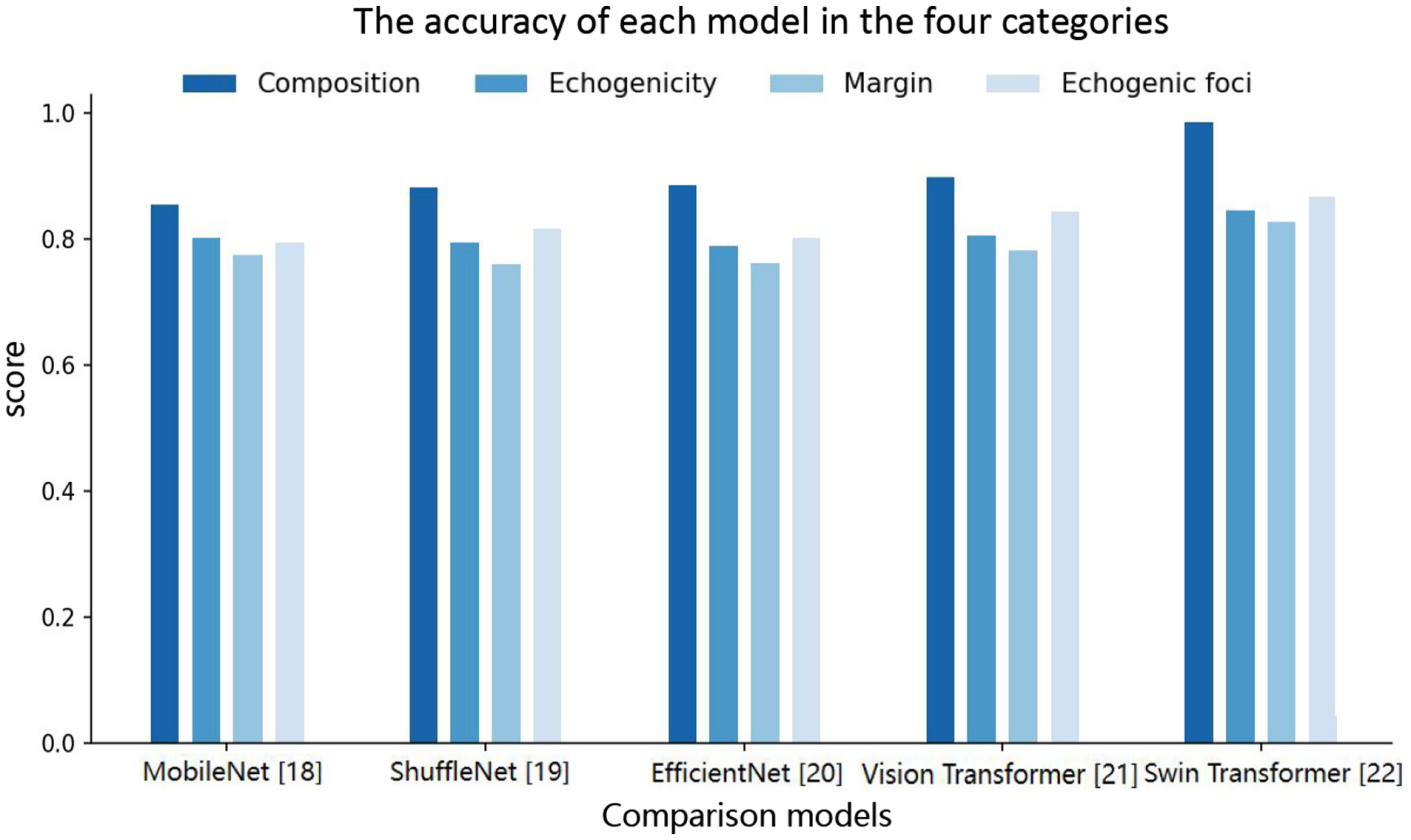
Comparison of classification models: performance of each model in different feature categories in the thyroid nodule classification task.

**Figure 5:**
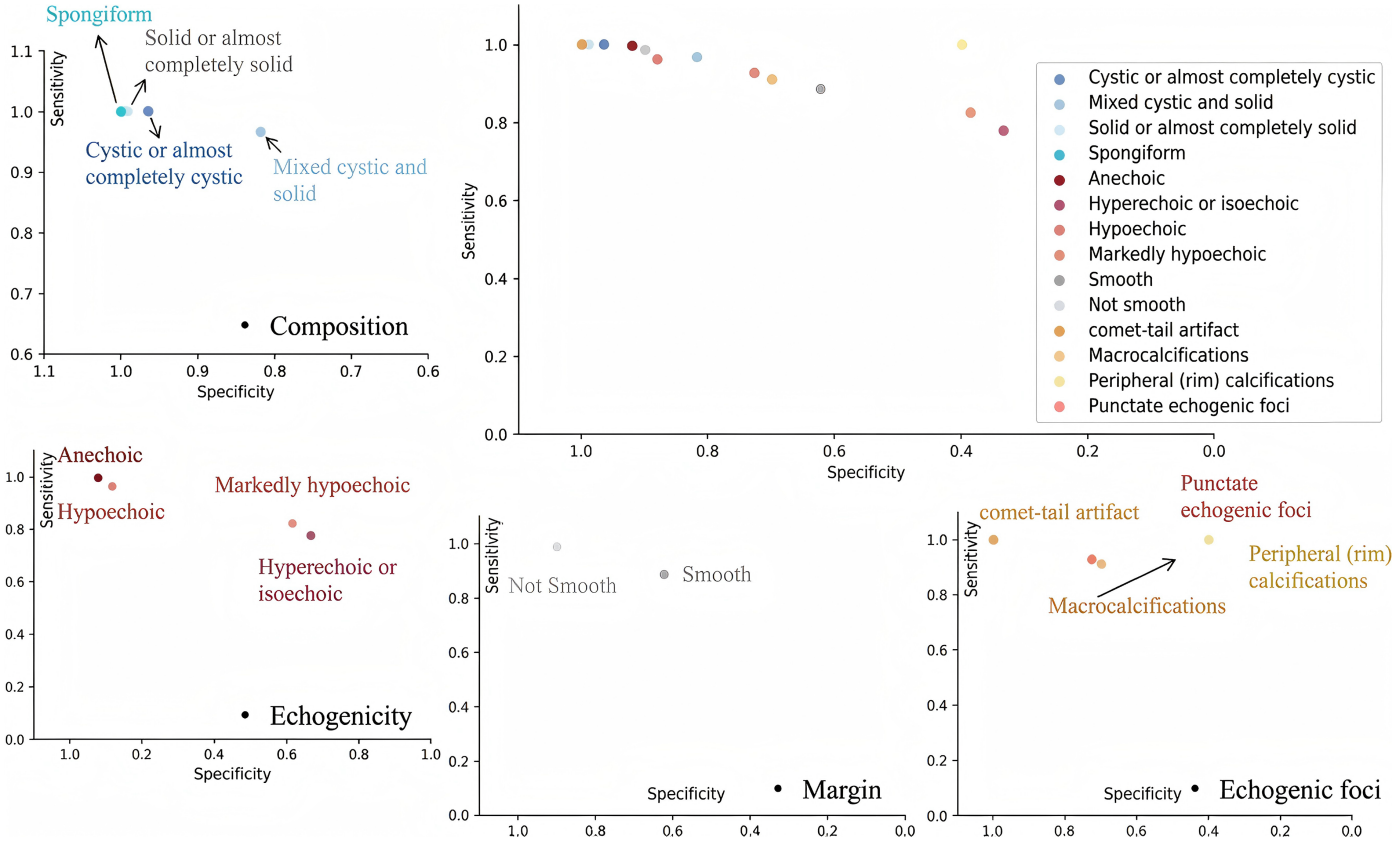
Experimental results of each class of Swin transformer: the Swin Transformer model classified five ultrasound features of thyroid nodules and demonstrated the sensitivity and specificity of different ultrasound features.

In an in-depth comparative analysis of recent mainstream classification models MobileNet (35), ShuffleNet (36), EfficientNet (37), Vision Transformer (38) and Swin Transformer (26), Figure 4 reveals the performance of each model in different feature categories in the thyroid nodule classification task. In particular, the Swin Transformer model shows excellent accuracy on the four key categories of nodules: Composition, Echogenicity, Margin and Echogenic foci, which are 0.985, 0.855, 0.826 and 0.877, respectively. These indicators not only highlight the high efficiency of Swin Transformer in capturing the subtle features of thyroid nodules, but also confirm its superiority in classification tasks. Based on these remarkable results, we selected Swin Transformer as the optimal classification model to further explore its performance in more detailed subcategories. Figure 5 will provide an in-depth analysis of Swin Transformer’s specific performance in each subcategory, thus providing a solid empirical basis for our model selection.

Figure 5 shows a comparison of the sensitivity and specificity of different ultrasound features in the diagnosis of thyroid nodules. In terms of composition, Spongiform nodules have higher sensitivity but lower specificity, while Solid or almost completely solid nodules show higher specificity. Echogenicity shows that anechoic and hypoechoic nodules have higher sensitivity but relatively lower specificity, while hyperechoic or isoechoic nodules have higher specificity. Nodules with rough margins have higher sensitivity, while smooth margins have higher specificity. Among echogenic foci, Punctate echogenic foci have higher specificity, and comet-tail artifacts have higher sensitivity and specificity, and are reliable diagnostic features. Different features have their own advantages and disadvantages in diagnosis. Features with high sensitivity are helpful for screening, while features with high specificity are helpful for diagnosis.

**Figure 6:**
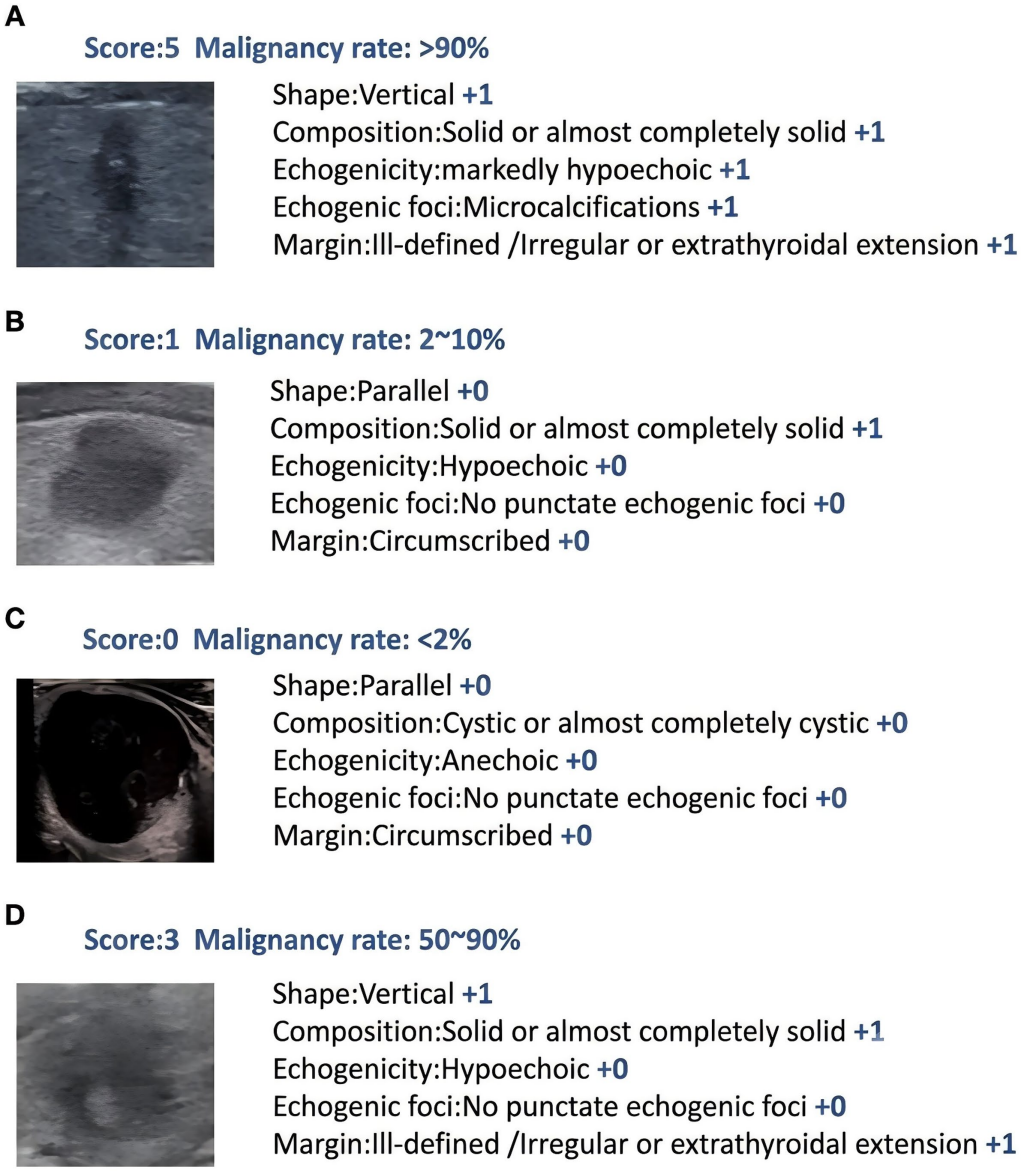
Experimental result: list some of the final output images of the model after nodule detection and classification.

Figure 6 illustrates the final output images of the model after nodule detection and classification. These images not only demonstrate the model’s detection and classification capabilities but also visually reflect the model’s scoring and discriminating performance on different types of nodules. Figure 6A shows a typical nodule with Ill-defined Irregular or extrathyroidal extension margins, classified as Not Smooth. After consultation with clinical experts, we simplified the edge characteristics into two categories: smooth and not smooth. The structure is classified as Solid, and the echogenicity as markedly hypoechoic. The final score for this nodule is 5, corresponding to a C-TIRADS classification of 5, indicating a highly suggestive malignant nodule. Figure 6B shows a typical Solid nodule, with its structure classified as Solid or almost completely solid. The final score for this nodule is 1, corresponding to a C-TIRADS classification of 4A, indicating a Low suspicion nodule. Figure 6C shows a nodule with a score of 0, a typical Anechoic nodule, indicating a benign nodule. Figure 6D shows a typical No punctate echogenic foci artifact. The final score for this nodule is 3, corresponding to a C-TIRADS classification of 4C, indicating a Highly suspicious nodule.

In the clinical validation phase, 303 representative ultrasound images corresponding to 303 thyroid nodules from 284 adult patients were used as the independent validation set for the AI model. The benign or malignant status of each nodule in the validation set was determined by surgical pathology or fine-needle aspiration cytology classified as Bethesda II or VI and was not derived from C-TIRADS scoring. The validation set included 94 benign nodules and 209 malignant nodules. To reduce potential identification bias, the images were randomly shuffled and renamed before physician assessment. Physicians independently evaluated the nodules using annotation software we developed, and subsequently conducted a second evaluation with the assistance of the AI model.

In the clinical validation set, the AI model achieved an overall accuracy of 0.862 [95% CI, 0.822–0.901]. The accuracy of unaided physicians was 0.705 [95% CI, 0.657–0.756]. With AI assistance, physician accuracy increased to 0.845 [95% CI, 0.802–0.884] (Table 2).

**Table 2:** Clinical validation results of the model: accuracy evaluation of the diagnostic performance of the model, physicians, and AI-assisted physicians in the test set.

		Precision	Recall	F1	Accuracy
Model	Benign	0.710 [95% CI: 0.631–0.797]	0.936 [95% CI: 0.886–0.979]	0.807 [95% CI: 0.747–0.861]	0.862 [95% CI: 0.822–0.901]
	Malignant	0.967 [95% CI: 0.939–0.994]	0.827 [95% CI: 0.777–0.878]	0.891 [95% CI: 0.858–0.924]	
Physician	Benign	0.515 [95% CI: 0.434–0.600]	0.738 [95% CI: 0.655–0.821]	0.606 [95% CI: 0.530–0.678]	0.705 [95% CI: 0.657–0.756]
	Malignant	0.852 [95% CI: 0.793–0.902]	0.689 [95% CI: 0.626–0.751]	0.761 [95% CI: 0.713–0.806]	
AI-assisted Physician	Benign	0.692 [95% CI: 0.606–0.769]	0.904 [95% CI: 0.839–0.957]	0.783 [95% CI: 0.721–0.837]	0.845 [95% CI: 0.802–0.884]
	Malignant	0.950 [95% CI: 0.918–0.978]	0.819 [95% CI: 0.767–0.870]	0.879 [95% CI: 0.844–0.911]	

For benign nodules, the AI model achieved a precision of 0.710 [95% CI, 0.631–0.797], recall of 0.936 [95% CI, 0.886–0.979], and F1 score of 0.807 [95% CI, 0.747–0.861]. The corresponding values for unaided physicians were 0.515 [95% CI, 0.434–0.600], 0.738 [95% CI, 0.655–0.821], and 0.606 [95% CI, 0.530–0.678], respectively. With AI assistance, physician performance for benign nodules was 0.692 [95% CI, 0.606–0.769] for precision, 0.904 [95% CI, 0.839–0.957] for recall, and 0.783 [95% CI, 0.721–0.837] for F1 score.

For malignant nodules, the AI model achieved a precision of 0.967 [95% CI, 0.939–0.994], recall of 0.827 [95% CI, 0.777–0.878], and F1 score of 0.891 [95% CI, 0.858–0.924]. Unaided physicians achieved a precision of 0.852 [95% CI, 0.793–0.902], recall of 0.689 [95% CI, 0.626–0.751], and F1 score of 0.761 [95% CI, 0.713–0.806]. With AI assistance, the corresponding values were 0.950 [95% CI, 0.918–0.978], 0.819 [95% CI, 0.767–0.870], and 0.879 [95% CI, 0.844–0.911], respectively.

These descriptive results suggest that AI assistance was associated with improved physician diagnostic performance in this exploratory validation set. However, because the AI-assisted reading was not fully blinded to model performance and comprehensive statistical comparisons were not available, these findings should be interpreted cautiously.

## 5 Discussion

The aim of this study was to develop a C-TIRADS-guided decision-support framework for thyroid nodule detection, ultrasound feature classification, and malignancy risk stratification. The proposed system was designed to standardize the assessment of key C-TIRADS ultrasound features and to provide a structured risk category rather than to replace histopathological diagnosis.

In the exploratory clinical validation set, AI assistance was associated with an increase in physician accuracy from 0.705 [95% CI, 0.657–0.756] to 0.845 [95% CI, 0.802–0.884]. These results suggest that the model may help physicians perform more standardized thyroid ultrasound assessments. However, given the limited size and class imbalance of the validation set, the findings should be interpreted as preliminary.

It is expected that the model of this study will bring many benefits in clinical application: First, the standardized diagnostic template provided by this model can simplify the diagnostic process and shorten the diagnostic time regardless of the physician’s experience. Secondly, the model is of great value in teaching and training, as it can provide young doctors with a standardized operating template and help them master the standardized process of thyroid ultrasound diagnosis. The proposed system may also have potential value for teaching and standardized reporting by providing a structured C-TIRADS-based assessment template. However, whether it can reduce unnecessary fine-needle aspiration biopsies or improve clinical workflow requires further prospective validation and decision-curve analysis.

Although the proposed model showed promising exploratory results, several limitations should be acknowledged, it still has some limitations. First, the clinical validation set was relatively small and imbalanced, with 94 benign and 209 malignant nodules. This distribution does not fully reflect the prevalence of malignancy in general thyroid nodule screening populations and may affect the generalizability of PPV, NPV, and overall clinical performance. Future validation should include larger and more balanced external cohorts from multiple centers. Second, the clinical validation design may have introduced expectation bias. In the AI-assisted reading phase, physicians were informed of the model performance before re-evaluating the images. In addition, only two physicians participated in the reader study, and a fully blinded multi-reader multi-case design with a predefined washout interval was not available. Therefore, the observed improvement in physician performance should be interpreted as exploratory. Third, the statistical analysis of the current study was limited by the available data. Because only binary classification results and aggregate diagnostic metrics were available, ROC analysis, calibration analysis, and decision-curve analysis were not performed. Therefore, AUC, calibration performance, and clinical net benefit could not be evaluated in this version. Future studies should preserve case-level probability outputs and perform comprehensive statistical analyses.

## 6 Conclusion

This study developed a C-TIRADS-guided decision-support framework for thyroid nodule detection, ultrasound feature classification, and malignancy risk stratification. The model first localized thyroid nodules and then classified key C-TIRADS features, including composition, echogenicity, shape, margin, and echogenic foci. The predicted feature scores were aggregated to generate a guideline-based risk category.

In the independent clinical validation set, the AI model achieved an overall accuracy of 0.862 [95% CI, 0.822–0.901]. Physician accuracy increased from 0.705 [95% CI, 0.657–0.756] without AI assistance to 0.845 [95% CI, 0.802–0.884] with AI assistance. These findings suggest that the proposed system may support standardized thyroid ultrasound interpretation. However, the model should not be interpreted as an independent substitute for histopathological diagnosis. Further blinded multicenter validation with larger and more balanced cohorts is required before routine clinical application.

## Data Availability

The datasets presented in this article are not readily available due to ethical concerns. Requests to access the datasets should be directed to the corresponding author’s email address.
